# Increasing gap junction coupling suppresses ibutilide-induced torsades de pointes

**DOI:** 10.3892/etm.2014.1601

**Published:** 2014-03-04

**Authors:** LEI RUAN, XIAOQING QUAN, LIANDONG LI, RONG BAI, MINGKE NI, RENDE XU, CUNTAI ZHANG

**Affiliations:** 1 Department of Gerontology, Tongji Hospital, Tongji Medical College, Huazhong University of Science and Technology, Wuhan, Hubei 430030, P.R. China; 2 Department of Cardiology, Beijing Anzhen Hospital, Capital Medical University, Beijing 100029, P.R. China

**Keywords:** long QT syndrome, wedge, ion channel, torsades de pointes

## Abstract

Drug-induced torsades de pointes (TdP) is common with class III antiarrhythmic drugs. Increased transmural dispersion of repolarization (TDR) contributes significantly to the development of TdP. Gap junctions play an important role in maintaining TDR in long QT syndrome. The present study examined the effect of a gap junction enhancer, antiarrhythmic peptide 10 (AAP10), on ibutilide-induced TdP. Coronary-perfused rabbit ventricular wedge preparations were used to evaluate the effect of AAP10 on ibutilide-induced arrhythmia. Transmural electrocardiograms and action potentials were recorded simultaneously. Early afterdepolarizations (EADs), R-on-T extrasystole, TdP and changes in Tpeak-end (Tp-e) and the Tp-e/QT ratio were observed. Changes in the levels of non-phosphorylated connexin 43 (Cx43) were measured by immunoblotting. Compared with those in the control group, the QT interval, Tp-e/QT and incidence rates of EAD and TdP increased with augmented dephosphorylation in the ventricular wedge preparations perfused with ibutilide under conditions of hypokalemia and hypomagnesemia. In the presence of AAP10, the incidence rates of EAD and TdP were reduced and the Tp-e/QT ratio decreased, with a parallel reduction in the level of non-phosphorylated Cx43. The results indicate that AAP10 suppressed ibutilide-induced TdP under conditions of hypokalemia and hypomagnesemia by decreasing TDR. AAP10 reduced TDR, possibly by preventing the dephosphorylation of Cx43 and thereby increasing myocardial cell gap junction coupling.

## Introduction

Acquired long QT syndrome (LQTS) is a potentially fatal medical condition that can be exacerbated by a wide range of antiarrhythmic drugs, particularly those in class III (1,2). Ibutilide is a new class III antiarrhythmic drug that functions to recover atrial flutter and atrial fibrillation, but may cause torsades de pointes (TdP). The drug works by prolonging the duration of the action potential, particularly under conditions of hypokalemia and hypomagnesemia (3,4). Ibutilide prolongs repolarization by enhancing the persistent Na^+^ current and suppressing the Ikr current (5).

Several studies have indicated that amplified transmural and transseptal dispersion of repolarization (TDR) is essential for the development of TdP in congenital and acquired LQTS (6–9). The amplified TDR exacerbates differential refractoriness across the myocardial wall, inducing early afterdepolarization (EAD) and R-on-T extrasystole, which initiates and maintains re-entrant circuits (10).

TDR is a result of significant heterogeneity in the expression of ion channels among various cell types in the ventricular wall (10). Such intrinsic electrophysiological heterogeneity is diminished due to the existence of gap junctions. Gap junctions permit the movement of small molecules along the electrochemical gradient and thus assist the electrical synchronization of adjacent myocytes, reducing TDR. Enhancing gap junction coupling is considered to result in the reduction of TDR and thus provide an antiarrhythmic effect, particularly in LQTS. Antiarrhythmic peptide 10 (AAP10) is a gap junction opener (11) that inhibits ventricular arrhythmia, particularly TdP, in various congenital LQTS models mimicked by drugs in animal experiments (12,13).

The aim of the present study was to determine whether AAP10 inhibits ibutilide-induced TdP, a type of drug-induced LQTS. In addition, the mechanism of TdP was explored with the aim of providing a safe method for the use of ibutilide.

## Materials and methods

### Study approval

All experiments involving animals were approved by the Institutional Animal Care and Use Committee of Tongji Medical College (Wuhan, China).

### Arterially perfused rabbit left ventricular wedge preparations

Arterially perfused rabbit left ventricular wedges were prepared by a standard technique. Japanese white rabbits, provided by the Wuhan Institute of Biological Products (Wuhan, China), were anesthetized with 35–40 mg/kg sodium pentobarbital (i.v.) and anticoagulated with heparin. The hearts were quickly excised and submerged in cold (4°C) cardioplegic solution (mmol/l): NaCl, 109; KCl, 24; NaH_2_PO_4_, 0.9; NaHCO_3_, 20; CaCl_2_, 1.8; MgSO_4_, 0.5; and glucose, 5.5. The left circumflex branch of the coronary artery was cannulated and perfused with the same cardioplegic solution. Unperfused areas of the left ventricle, easily identified by the reddish appearance due to the existence of unflushed erythrocytes, were removed.

### Electrophysiological observations of the wedge preparations

Cannulated preparations were placed in a small heated tissue bath and arterially perfused with Tyrode’s solution (mmol/l): NaCl, 129.0; KCl, 4.0; NaH_2_PO_4_, 0.9; NaHCO_3_, 20; CaCl_2_, 1.8; MgSO_4_, 0.5; and glucose, 5.5; buffered with 95% O_2_ and 5% CO_2_. The temperature was maintained at 35.7±0.2°C and the perfusion pressure was maintained at 35–45 mmHg by a peristaltic pump. A bipolar concentric silver electrode fixed to the surface of the endocardium provided a continuous single stimulus with a basic cycle length (BCL) of 2,000 msec. Two Ag/AgCl electrodes were placed on the two sides of the wedge and were used to record the pseudo-electrocardiograms. Transmembrane action potentials were recorded simultaneously by floating glass microelectrodes (direct current resistance, 10–20 MΩ) filled with 2.7 M KCl.

### Study protocols

Japanese white rabbits (weight, 2–2.5 kg) of either gender, were randomly divided into the five following groups: Control, hypokalemia and hypomagnesemia (hypo), ibutilide, ibutilide and hypo and AAP10 groups. The control group (n=9) were perfused with Tyrode’s solution. The hypo group (n=9) were perfused with a modified reduced-potassium and reduced-magnesium Tyrode’s solution (mmol/l): NaCl, 131.0; KCl, 2.0; NaH_2_PO_4_, 0.9; NaHCO_3_, 20; CaCl_2_, 1.8; MgSO_4_, 0.25; and glucose, 5.5. The ibutilide group (n=9) were perfused with Tyrode’s solution for 1 h, which was followed by the addition of 2 mg/l ibutilide (Anhui BBCA Pharmaceutical Co., Ltd., Anhui, China). The development of spontaneous and programmed electrical stimulation (PES)-induced TdP was observed, as well as the EAD and changes in the QT interval and Tp-e. The ibutilide and hypo group (n=9) were perfused with ibutilide dissolved in modified reduced-potassium and reduced-magnesium Tyrode’s solution. The AAP10 group (n=9) were perfused with 500 nM AAP10 (Chinese Peptide Co., Hangzhou, China) for 15 min, which was followed by the addition of a solution containing 500 nM AAP10 and 2 mg/l ibutilide in modified reduced-potassium and reduced-magnesium Tyrode’s solution.

The QT interval was defined as the time from the onset of QRS to the point at which the final downslope of the T wave crossed the isoelectric line. TDR was measured by Tp-e (from the peak to the end of the T wave). The Tp-e and QT interval ratio were measured in the same beats.

### Western blotting

The ventricular wedge preparations were removed from the tissue bath following the completion of PES and immediately frozen at −80°C. The frozen tissues were pulverized with a mortar and pestle that had been cooled in liquid nitrogen. The samples were then homogenized in a lysis buffer containing 30 mM Tris (pH 7.4), 150 mM NaCl, 1% nonylphenoxypolyethoxyethanol, 0.25% sodium deoxycholate, 1 mM EDTA, 0.1 mM phenylmethylsulfonyl fluoride, 1 μg/ml aprotinin, 1 μg/ml pepstatin and 1 μg/ml leupeptin. Homogenates were cleared by centrifugation at 10,000 × g for 30 min at 4°C.

Lysate samples containing 50 μg total protein were resolved by sodium dodecyl sulfate-polyacrylamide gel electrophoresis and immunoblotted. GAPDH protein was used as a control to ensure equal protein loading. Primary antibody incubations were performed overnight at 4°C using mouse monoclonal antibodies to measure the non-phosphorylated component of connexin 43 or total connexin 43 (Cx43; 1:1,000 dilution; Zymed Laboratories, Inc., San Francisco, CA, USA). After washing, the membranes were incubated with horseradish peroxidase-conjugated goat anti-mouse secondary antibodies (1:1,000 dilution; Novogen, Inc., Madison, WI, USA), treated with chemiluminescence reagent (Pierce Biotechnology, Inc., Rockford, IL, USA) and exposed to X-ray film. Immunoreactivity was quantified by densitometric analysis with Image-Pro Plus 6.0 software (Media Cybernetics Inc., Rockville, MD, USA). The quantity of Cx43 was defined by the band density corresponding to the Cx43 protein normalized against GAPDH.

### Statistical analysis

Statistical analysis was performed using the Student’s t-test or one-way analysis of variance. Fisher’s exact test was used for comparing event incidences, including the occurrence of EAD, R-on-T extrasystole and TdP. All values are expressed as mean ± SEM, unless otherwise noted. P<0.05 was considered to indicate a statistically significant difference. Statistical analyses were performed with SPSS for Windows (Version 13.0, SPSS, Inc., Chicago, IL, USA).

## Results

### Effect of ibutilide on cardiac electrophysiological parameters

As with other class III antiarrhythmic drugs, the action potential and QT interval were significantly prolonged by ibutilide at a BCL of 2,000 msec. In the ibutilide group, the QT interval was increased from 303±19 to 498±68 msec (P<0.001, vs. control), Tp-e was increased from 50±7 to 116±24 msec (P<0.01, vs. control) and the Tp-e/QT ratio was increased from 0.16±0.02 to 0.23±0.04 (P<0.01, vs. control). The results show that ibutilide markedly increased the Tp-e interval compared with the QT interval (Table I).

The incidence of EAD was 3/9 (P=0.10, vs. control) and was accompanied by R-on-T extrasystole. No spontaneous TdP or other ventricular arrhythmias were observed in the ibutilide group (Table II).

### Changes in electrophysiological parameters in the ibutilide and hypo group

When ibutilide was perfused under conditions of hypokalemia and hypomagnesemia with a BCL of 2,000 msec, the prolongation of the QT interval was greater than that with ibutilide alone. The QT interval extended to 611±168 msec (P<0.01, vs. ibutilide group), while Tp-e was also extended to 190±79 msec (P<0.001, vs. ibutilide group). The Tp-e/QT ratio increased to 0.31±0.08 (P<0.01, vs. ibutilide group; Table I).

**Figure 1:**
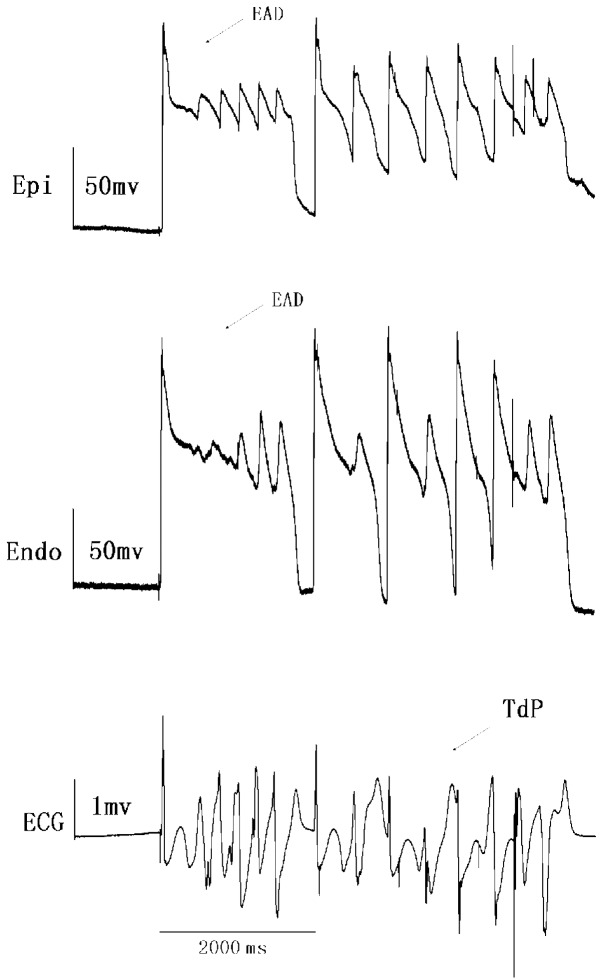
Spontaneous TdP in the ibutilide and hypo group. Following PES, long interval spontaneous EAD and TdP are generated by stimulation. The EAD conducted across the ventricle induces re-entry. TdP, torsades de pointes; EAD, early afterdepolarization; PES, programmed electrical stimulation; hypo, hypokalemia and hypomagnesemia.

**Figure 2:**
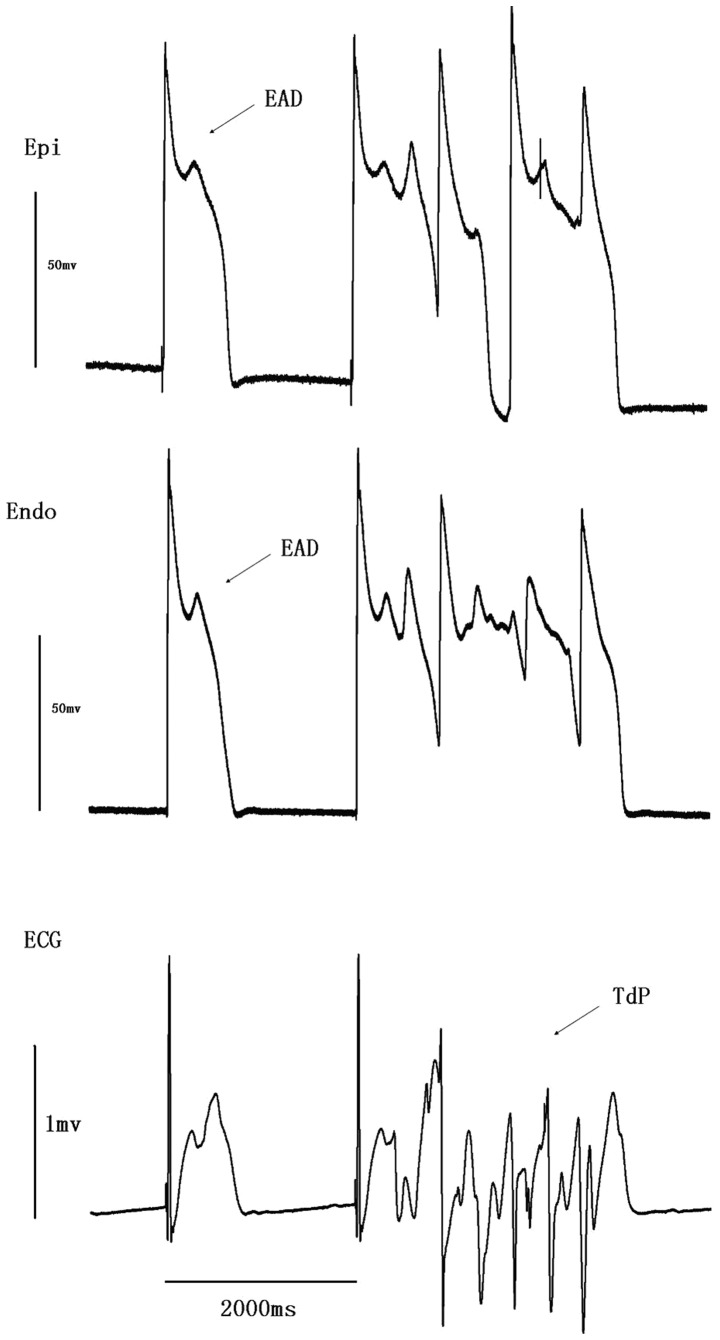
EAD-triggered activity generated in endocardial cells in the ibutilide and hypo group. The preparation was paced from the endocardial surface at a BCL of 2,000 msec. Spontaneous EAD and triggered activity was generated in endocardial cells. BCL, basic cycle length; EAD, early afterdepolarization; hypo, hypokalemia and hypomagnesemia.

EAD was observed in four preparations (n=9) while TdP occurred in seven (P<0.01, vs. ibutilide group; Table II). TdP in four preparations was spontaneous (Fig. 1 and 2), while in the other three preparations TdP was induced by PES. Spontaneous TdP was always followed by EAD and R-on-T extrasytole.

### Effect of AAP10 on the QT interval, Tp-e and the incidence of EAD, R-on-T extrasystole and TdP

**Figure 3:**
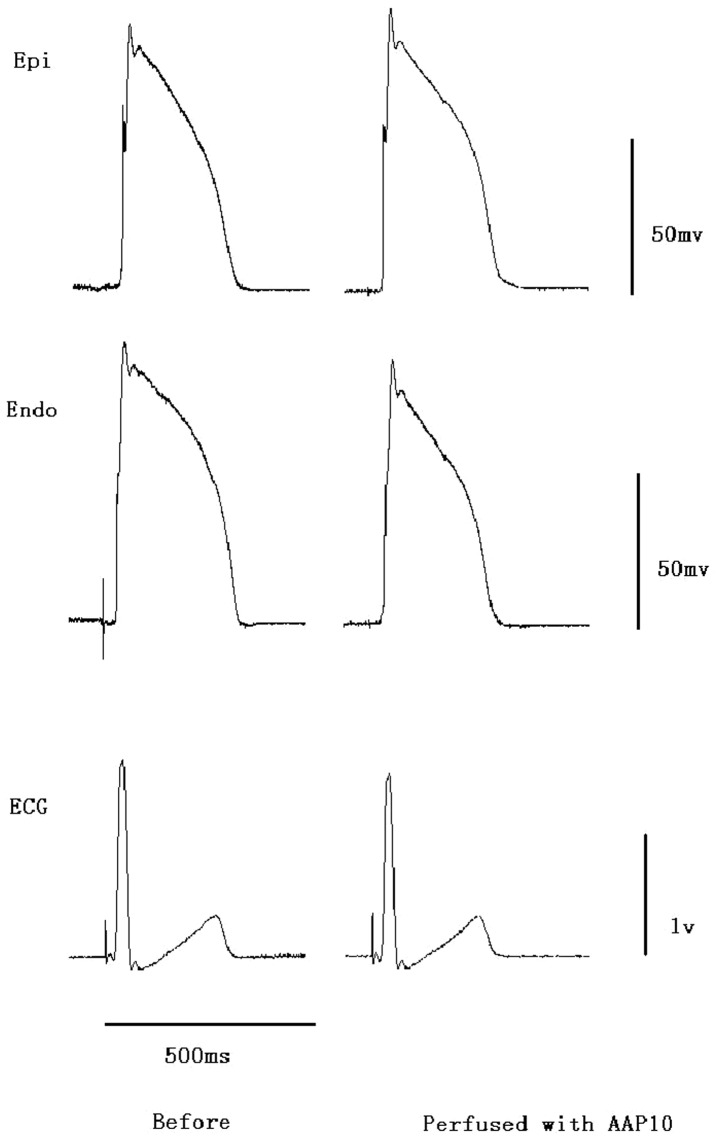
Perfusion with AAP10 at a concentration of 500 nM did not significantly influence the QT interval, action potential duration, Tp-e or QRS duration. AAP10, antiarrhythmic peptide 10.

**Figure 4:**
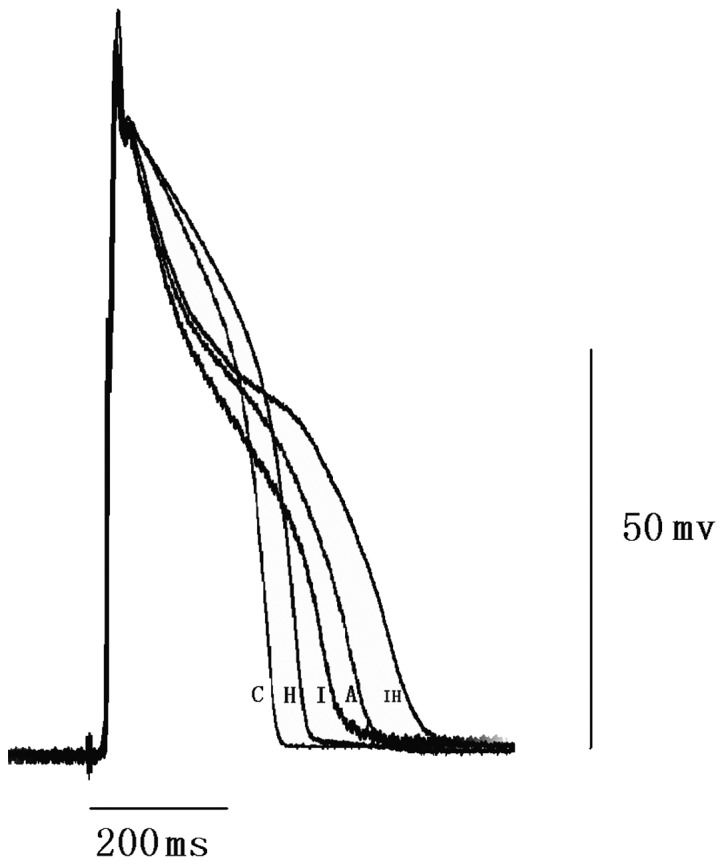
Action potentials recorded from epicardial region sites of arterially perfused rabbit left ventricular wedge preparations in various groups. Action potential duration was increased by ibutilide and hypopotassium and hypomagnesium Tyrode’s solution. However, AAP10 reversed the conditions and shortened the action potential duration. C, control group; H, hypo group; I, ibutilide group; A, AAP10 group; IH, ibutilide and hypo group; AAP10, antiarrythmic peptide 10; hypo, hypokalemia and hypomagnesemia.

Prior to the administration of ibutilide, perfusion with AAP10 at a concentration of 500 nM did not significantly affect the QT interval, action potential duration, Tp-e or QRS duration (Fig. 3). However, the prolonged action potentials, QT intervals and Tp-e induced by ibutilide under conditions of hypokalemia and hypomagnesemia were reversed by AAP10 (Fig. 4 and Table I). The QT interval decreased to 459±52 msec (P<0.01, vs. ibutilide and hypo group), Tp-e decreased to 93±31 msec (P<0.001, vs. ibutilide and hypo group) and Tp-e/QT decreased to 0.20±0.05 (P<0.001, vs. ibutilide and hypo group). The reduction in Tp-e/QT showed that the reduction in Tp-e was greater that than in the QT interval.

EAD and R-on-T extrasystole were observed in 3 preparations (n=9), while TdP occurred in 1 preparation (P<0.01, vs. ibutilide and hypo group; Table II).

### Changes in the phosphorylation levels of Cx43 serine 368

**Figure 5:**
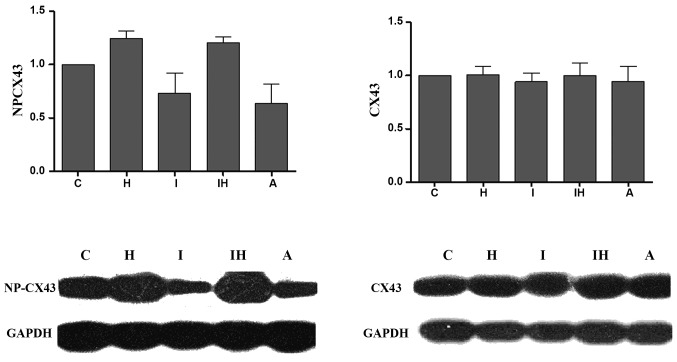
Western blotting results and quantitative densitometric analysis of non-phosphorylated Cx43 and total Cx43. Signals were quantified by densitometry and normalized against GAPDH. There are no significant differences among the control and other groups for total Cx43. However, the non-phosphorylated Cx43 level is significantly increased in the ibutilide and hypo group (P<0.05, vs. control). AAP10 at a concentration of 500 nM significantly attenuated the increase in non-phosphorylated Cx43 (P<0.05, vs. ibutilide and hypo group). C, control group; H, hypo group; I, ibutilide group; A, AAP10 group; IH, ibutilide and hypo group; AAP10, antiarrythmic peptide 10; hypo, hypokalemia and hypomagnesemia; Cx43, connexin 43.

Comparisons between the serine 368 non-phosphorylated Cx43 and total Cx43 expression profiles in the various groups are shown in Fig. 5. There were no significant differences in total Cx43 expression levels among the various groups. The level of Cx43 with non-phosphorylated serine 368 increased in the ibutilide and hypo group compared with that in the control group. This indicates that the majority of serine 368 residues in Cx43 molecules localized in gap junctions in the normal myocardium are phosphorylated. AAP10 at 500 nM attenuated the increase in non-phosphorylated residues in the ibutilide and hypo group. This indicates that AAP10 can reverse the dephosphorylation of serine 368 in Cx43, which is consistent with AAP10 reducing the incidence of arrhythmia.

## Discussion

Prolonging the QT interval with drugs can result in a predisposition to TdP, which may degenerate into ventricular fibrillation and cause sudden mortality (14). Drug-induced TdP may be more common than initially hypothesized (15). A number of medicines lead to drug-induced TdP, including class IA and III antiarrhythmic drugs, antibiotics and antidepressants (16).

Atrial fibrillation is one of the most common arrhythmias, particularly with increasing age. The incidence has been shown to reach 10% among individuals >80 years-old (17). Ibutilide is a new class III antiarrhythmic drug that has been widely used in recent years to treat cardioversion atrial fibrillation and atrial flutter in clinical practice. The cardioversion rate with ibutilide is significantly higher than with other antiarrhythmic drugs, particularly for atrial flutter cardioversion (18). However, several clinical trials have reported adverse effects, including non-sustained and sustained polymorphic ventricular tachycardia (VT). A meta-analysis of five studies found the occurrence of TdP with ibutilide to be 1.7% and the incidence of polymorphic VT with ibutilide to be higher than with sotalol (3). Thus, in-hospital cardiac monitoring is recommended when ibutilide infusion is initiated (1).

Animal studies have indicated that ibutilide produces a greater degree of TDR, as well as a higher incidence of EAD and TdP in cardiomyopathic dogs compared with acute atrioventricular block (19). The present study identified that Tp-e and Tp-e/QT also increased in normal rabbit wedge preparations when perfused with ibutilide. The incidence of TdP increased under conditions of hypokalemia and hypomagnesemia, a characteristic of drug-induced LQTS.

The pharmacological effects of ibutilide are Ikr-blockade and persistent Na^+^ current activation. However, the heterogeneity of Ikr distribution and the persistent Na^+^ current channels between myocardial cells leads to an increased TDR across the ventricular wall. Several studies have indicated that amplified TDR is the primary basis of the mechanism essential for the development of TdP (20–23). The increased TDR creates a vulnerable window for the development of re-entry. The reduction in net repolarizing current also results in a predisposition to developing EAD-induced triggered activity in myocardial cells, which provides the extrasystole that triggers TdP when it falls within the vulnerable window (24–26). The studies by Yan et al (24–26) and a number of previous studies suggest that Tp-e/QT disturbance may be a new index for predicting heart arrhythmia (27–31). This method is superior to TDR, to a certain extent, since it corrects the impact of the QT interval for TDR.

In the present study, the Tp-e/QT ratio was increased by ibutilide resulting in TdP. AAP10 was found to decrease Tp-e/QT and suppress ibutilide-induced TdP. Our studies have shown that gap junctions play an important role in TDR (12–13). Through opening gap junctions the TDR is reduced to a certain extent, resulting in enhanced ion exchange between cells. In the model of congenital LQTS mimicked by drugs, increasing the coupling of gap junctions resulted in the inhibition of TdP and the reduction of TDR, through strengthening the ion exchange between various cells (12,13).

Gap junctions are electrical and chemical channels located between adjacent myocardial cells and are the major constituent of intercalated discs. The main gap junction protein in ventricular muscle is Cx43. AAP10 is a synthetic peptide with antiarrhythmic effects that functions by opening gap junctions (32). The coupling and uncoupling of gap junctions is primarily regulated by the phosphorylation of Cx43 (33). AAP10 binds to a G-protein-dependent membrane receptor and enhances the phosphorylation of serine 368 in Cx43 by activating protein kinase C (34). In the present study, the immunoblotting results revealed that the expression levels of non-phosphorylated Cx43 were much higher in the ibutilide and hypo group compared with the control. However, in the presence of AAP10, the dephosphorylation of serine 368 was decreased, which corresponds with reductions in Tp-e and Tp-e/QT and the incidence of arrhythmia. These results indicate that well-coupled gap junctions may function as a brake to limit increases in TDR under conditions of LQTS.

The present study indicated that AAP10 decreases the incidence of ibutilide-induced VT. The combined application of antiarrhythmic drugs has become increasingly studied in recent years. By balancing the various pharmacological mechanisms of antiarrhythmic drugs, the combined application of antiarrhythmic drugs may well offset side-effects, including proarrhthmia, without affecting the antiarrhythmic function.

The present study demonstrated that the gap junction enhancer, AAP10, significantly reduces Tp-e and Tp-e/QT and prevents TdP by inhibiting the dephosphorylation of Cx43 in ibutilide-induced LQTS. The study also indicated that the administration of AAP10 with a class III antiarrhythmic drug may be a novel approach for the treatment of arrhythmias while avoiding proarrhythmia.

## Figures and Tables

**Table I:** Changes in the electophysiological parameters of the various groups (n=9 per group).

Groups	QT interval msec	Tp-e msec	Tp-e/QT
Control	303±19^b^	50±7^b^	0.16±0.02^b^
Hypo	318±27^b^	62±15^b^	0.19±0.04^c^
Ibutilide	498±68^a^	116±24^b^	0.23±0.04^a^
Ibutilide and hypo	611±168	190±79	0.31±0.08
AAP10	459±52^a^	93±31^b^	0.20±0.05^b^

a P<0.01 and

b P<0.001, vs. the ibutilide and hypo group. The basic cycle length was 2,000 msec. Values are expressed as the mean ± SEM.

AAP10, antiarrhythmic peptide 10; Hypo, hypokalemia and hypomagnesemia.

**Table II:** Incidence of arrhythmia in the various groups (n=9 per group).

			TdP		
			
Groups	EAD	R-on-T	SPO	PES	Total
Control	0	0	0	0	0
Hypo	0	0	0	2	2
Ibutilide	3	3	0	0	0
Ibutilide and hypo	4	4	4	3	7
AAP10	3	3	1	0	1^a^

a P<0.01, vs. the ibutilide and hypo group.

PES, programmed electrical stimulation; SPO, spontaneous; EAD, early afterdepolarization; Hypo, hypokalemia and hypomagnesemia; AAP10, antiarrhythmic peptide 10; TdP, torsades de pointes.
